# Dynamic Sensing of Localized Corrosion at the Metal/Solution Interface

**DOI:** 10.3390/s120404962

**Published:** 2012-04-18

**Authors:** Wei Li, Boyu Yuan, Chao Wang, Liang Li, Shenhao Chen

**Affiliations:** 1 School of Mechanical and Electrical Engineering, China University of Mining and Technology, Xuzhou 221116, China; 2 School of Physics and Electronics Engineering, Jiangsu Normal University, Xuzhou 221116, China; 3 Department of Chemistry, Jiangsu Normal University, Xuzhou 221116, China; 4 Department of Chemistry, Shandong University, Jinan 250100, China

**Keywords:** localized corrosion, interfaces, Mach-Zehnder interferometer

## Abstract

A Mach-Zehnder interferometer is employed to detect localized corrosion at the metal/solution interface in the potentiodynamic sweep of the iron electrode in solutions. During the electrochemical reactions, local variations of the electrolyte's refractive index, which correlate with the concentration of dissolved species, change the optical path length (OPL) of the object beam when the beam passes through the electrolyte. The distribution of the OPL difference was obtained to present the concentration change of the metal ions visually, which enable direct evidence of corrosion processes. The OPL difference distribution shows localized and general corrosion during the anodic dissolution of the iron electrode in solutions with and without chloride ions, respectively. This method provides an approach for dynamic detection of localized corrosion at the metal/solution interface.

## 1. Introduction

Localized corrosion, a serious problem in industries for it often causes catastrophic failures in engineering structures, normally results from the breakdown of passive films in aggressive environments, especially in media containing chloride ions. Localized corrosion includes various types of corrosion phenomena such as pitting, crevice corrosion, stress corrosion cracking and corrosion fatigue [1]. In contrast to general corrosion, whose rate will normally be predictable from vast experience, localized corrosion tends to proceed at a high rate of metal dissolution from the local area. The investigation of localized corrosion is of particular interest and importance in many scientific and technological applications [2–11]. Although a lot of effort has been made to understand its mechanisms and to prevent its occurrence, localized corrosion is still a common cause of failures in seawater desalination units, gas and oil refining equipment, water-cooling systems and steel reinforced concrete structures [12].

Many techniques have been used to study the localized corrosion caused by chloride ions, most of which are involved direct current (DC) polarization [13,14], electrochemical noise (EN) technique [15,16], X-ray spectroscopy [17–19], electrochemical impedance spectroscopy (EIS) [19–22], scanning electrochemical microscopy (SECM) [23–25] and scanning vibrating electrode technique (SVET) [26–28]. DC polarization is probably the most used electrochemical technique in corrosion, where the majority of the achievements on localized corrosion were obtained. In recent decades the others were usually utilized in combination with DC polarization to study localized corrosion. All of these techniques have yielded considerable insights into localized corrosion processes. As the dynamic processes are not fully understood, more experimental methods are required for *in situ* observation of localized corrosion at the metal/solution interface.

Optical interferometry can provide quantitative measurement of the optical path length (OPL) distribution that allows transparent samples to be described with a diffraction-limited transverse resolution and a sub-wavelength axial accuracy [29,30]. The main advantages of this full-field optical method are: nondestructive working principle, fast response and the advanced performance of the systems [31]. With those merits, corrosion scientists have paid much attention to develop and utilize optical interferometric methods to study localized corrosion processes [6,32–34]. Punckt *et al*. employed ellipsomicroscopy for surface imaging (EMSI) and specially adapted high resolution contrast-enhanced optical microscopy to visualize the onset of pitting corrosion directly in the solution [6]. Habib used holographic interferometry to study the local corrosion of carbon steel in seawater [32]. Wang explored the digital speckle pattern interferometry to investigate pitting corrosion processes of metal [33]. Tada applied Mach-Zehnder interferometry and shadowgraphy to investigate the concentration field of Zn^2+^ during galvanic corrosion of a Zn/Steel couple successfully [34]. *In situ* observations have been carried out in our laboratory using digital holographic methods to study the dynamic processes of electrochemical reactions [10,11,35–40]. It has been proved to be effective in investigating the dynamic processes of the electrochemical reactions at the electrode/electrolyte interface.

In this paper, a Mach-Zehnder interferometer is employed to visualize localized corrosion processes at the metal/solution interface during anodic dissolution of iron in Na_2_SO_4_ and NaCl solutions in combination with linear sweep voltammetry. During the electrochemical processes of the iron electrode in the presence of chloride ions, the local area of the electrode dissolved as metal ions firstly, which led to high local concentration of the solution at the area. The distributions of the OPL difference are presented to observe the processes visually.

## 2. Experimental

### Voltammetric Measurements

**Figure 1.**
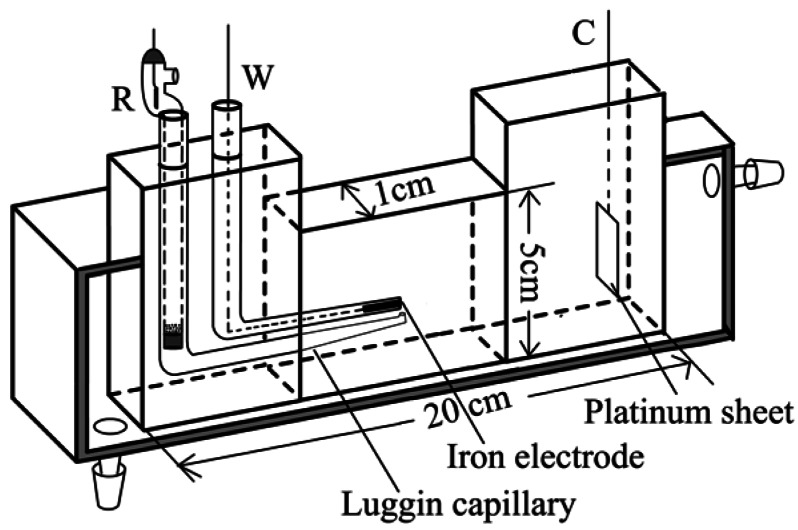
Schematic diagram of the electrochemical cell. R, reference electrode; W, working electrode; C, counter electrode.

As shown in Figure 1, the electrochemical cell contains a three-electrode system. The cell is made up of optical glass and its volume is about 100 mL. The iron electrode (Puratronic, Alfa Aesar, 99.99%) with a diameter of 2 mm served as the working electrode. It was sealed in a glass tube, with one end of tube sanded flat to expose the electrode. The counter electrode was a large sheet of platinum (0.8 cm × 2.8 cm). The reference electrode was a saturated calomel electrode (SCE) with a Luggin capillary, whose end is set near the surface of the iron electrode. All potentials reported here were with respect to SCE. Before each experiment, the iron electrode was mechanically abraded with #1500 and #3000 emery papers to a mirror-like brightness and then cleaned by alcohol and three triply distilled water in an ultrasonic bath. The electrolytes were 0.5 M NaCl solution and 0.5 M Na_2_SO_4_ solution. All solutions were prepared from reagents of analytical grade and triply distilled water. Voltammetric measurements were performed by means of CHI660B electrochemical station at room temperature. Prior to potentiodynamic polarization experiments, the open circuit potential (OCP) was recorded for 10 min. The positive sweep of potential was carried out at the rate of 10 mV·s^−1^ in each experiment.

### Optical system

**Figure 2.**
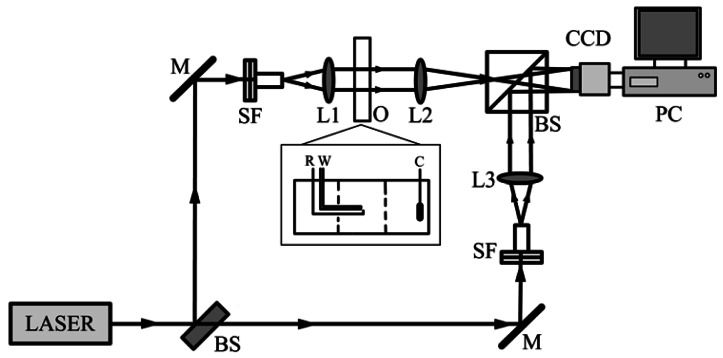
Experimental setup of the Mach-Zehnder interferometer. M, mirror; BS, beam splitter; SF, spatial filter; L1, L2 and L3, lens; O, the electrochemical cell.

**Figure 3.**
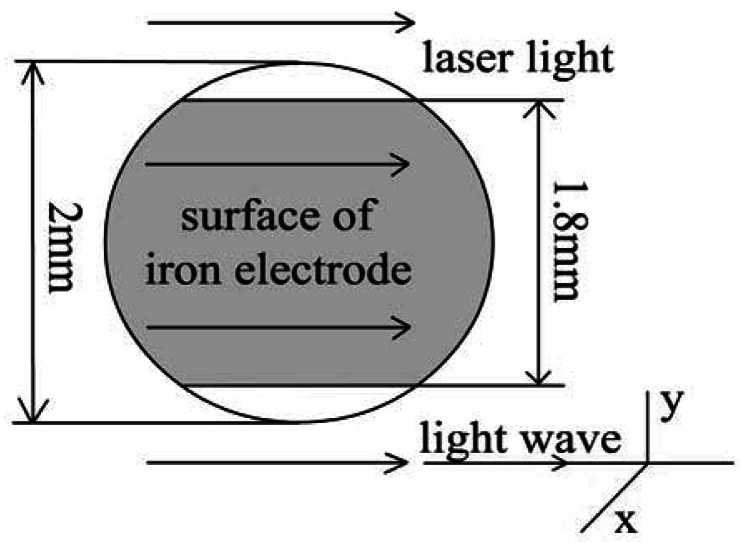
The object wave passing though the electrode surface. The *X* axis is in horizontal direction from the electrode surface toward the bulk electrolyte while the *Y* axis in vertical direction is parallel to the surface of electrode.

The experimental setup of the Mach-Zehnder interferometer is illustrated in Figure 2. The beam of a He-Ne laser with a wavelength of 632.8 nm was split into two beams, a reference wave and an object wave by a beam-splitter. Each beam was enlarged to a diameter of 50 mm by a beam expander including a spatial filter. The beam that carried the information of the metal/solution was combined with the reference wave by a beam-splitter cube and then recorded by a CCD sensor. A digital camera, the Sony DSR-PD150P, was used to record the dynamical process. It has a 1/3 inch sensor which can provide a progressive scanning format with 720 × 576 effective pixels. The interferograms corresponding with various states of the metal/solution interface were imaged by the camera. Ulead VideoStudio 8.0 software was used to capture the video signal. The videos were stored in the computer as AVI (audio visual interleaved) files at up to 25 frames per seconds. The distributions of the OPL difference at any intervals in the dynamic process can be obtained from the corresponding interferograms by various phase abstraction methods. In our measurement, Fourier method was employed to obtain the OPL information from the interferograms so as to achieve consecutive calculation and reduce the noise of the system. Since the object wave hits a circular sample from the side, the observed area (the gray area) is a changing sample area, as shown in Figure 3. It has been corrected during image processing.

## 3. Mathematical Model

During the electrochemical reactions, the anodic dissolution of metals resulted in the increase of concentration of the metal ions, which leaded to a refractive index variation of the solution at the metal/solution interface. The present experiment is based on the fact that the changes of the refractive index in the solution caused by the electrochemical reactions are associated with the OPL of the object wave when it goes through the solution.

If *n*_2_ and *n*_1_ are used to denote the refractive index after and before the reaction in the Mach-Zehnder interferometer, then [41]:

(1) $$\Delta L_{\left(x,y\right)}=\int \text{(}n_{2}-n_{1})\text{d}l=\int \Delta n_{\left(x,y\right)}\text{d}l$$

where *ΔL_(x,y)_* means the difference of the OPL of the object wave; *l* means the distance where the object wave passes through.

*ΔL_(x,y)_* can be obtained by the phase difference of the object wave [42]:

(2) $$\Delta L_{\left(x,y\right)}=[\Delta \phi_{\left(x,y\right)}/2\pi ]\lambda$$

where *ΔØ_(x,y)_* means the phase difference of the object wave; *λ* is the wavelength of the laser used.

In our measurement, Fourier analysis method is employed to extract *ΔØ_(x,y)_* from the interferograms. The recording is made with identical conditions of illumination, the intensity *I_(x, y, t,)_* recorded on the hologram at time *t* during the reactions is given by [36,43]:

(3) $$I(x,y,t)=a(x,y)+b(x,y)\cos[2\pi f_{0}x+\phi (x,y,t)]$$

where *a(x, y)* and *b(x, y)* are the background and modulation terms, respectively; *f_0_* is a spatial carrier frequency and the phase *Ø(x,y,t)* contains the desired information at different time. It can be also written in the following form:

(4) $$I(x,y,t)=a(x,y)+\frac{1}{2}b(x,y)\text{exp}i[2\pi f_{0}x+\phi (x,y,t)]+\frac{1}{2}b(x,y)\text{exp}\{-i[2\pi f_{0}x+\phi (x,y,t)]\}$$

A Gaussian band-pass filter was applied to obtain the phase distribution. After taken two-dimensional Fourier transform, band-pass filtering and inverse Fourier transform the image *I(x, y, t,)*′ can be written in the following form:

(5) $$I{\left(x,y,t\right)}^{'}=\frac{1}{2}b(x,y)\text{exp}i[2\pi f_{0}x+\phi (x,y,t)]$$

Then, the phase difference *Ø(x,y)* between the any time points *t_1_* and *t_2_* could be calculated by [43],

(6) Δϕ(x,y)=arctan{Im[I(x,y,t1)′*I(x,y,t2)′]Re[I(x,y,t1)′*I(x,y,t2)′]}

Generally, the phase change between two frames (0.04 second interval) is less than 2π. Meanwhile, phase differences greater than 2π give rise to an indeterminacy that can be resolved by use of standard phase-unwrapping methods. In the experiment, the minimal phase difference detected effectively was about 0.1 rad. The distribution of the difference of the OPL can be obtained from Equation 2 and the minimal difference of the OPL is about 10 nm.

## 4. Results and Discussion

During the electrochemical reactions, the concentration of iron ions might change with the time. The OPL difference *ΔL_(x,y)_* obtained from the interferograms, which is the sum of the OPL difference caused by the soluble species dissolved from the iron electrode, can sense the change of dynamic concentration changes and display the occurrence of corrosion processes of the electrode during the reactions. The area in which the OPL changes indicates the location at which the corrosion occurs. The distributions of the OPL difference *ΔL_(x,y)_* can reflect the corrosion processes during any intervals.

**Figure 4.**
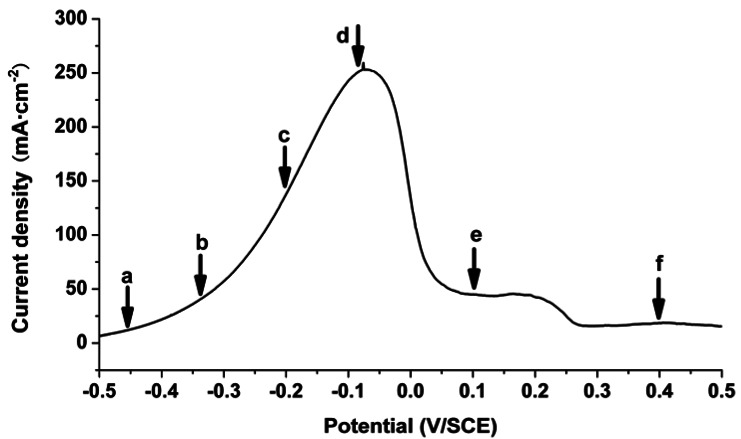
Potentiodynamic polarization curve of the iron electrode in 0.5 M Na_2_SO_4_ solution at 10 mV·s^−1^.

Figure 4 shows potentiodynamic sweep curve of iron in 0.5 M Na_2_SO_4_ solution at 10 mV·s^−1^. It is widely accepted that general corrosion occurs during the polarization processes of Fe/0.5 M Na_2_SO_4_ system. As illustrated in Figure 4, the current increases quickly with the increase in potential at the initial, and reaches a peak when the film is finally formed on the surface of the electrode. After the current peak, the iron electrode enters the passive region.

**Figure 5.**
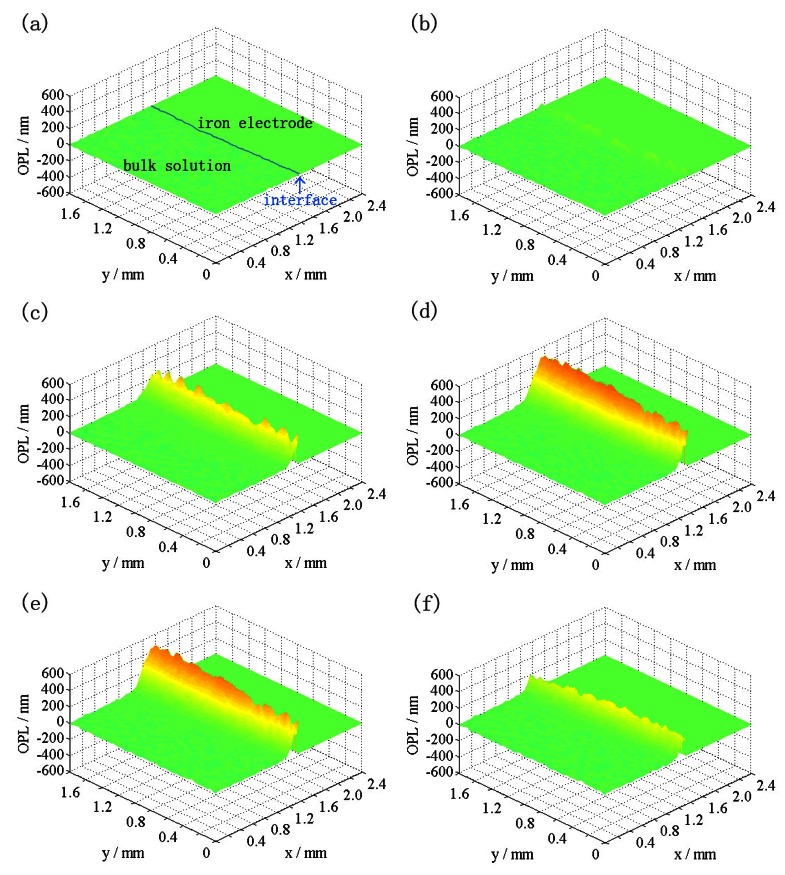
The distributions of the OPL difference at different times in comparison with the initial time during the process of the iron electrode in 0.5 M Na_2_SO_4_ solution at 10 mV·s^−1^.

The distribution of the OPL difference in comparison with the starting time during the reaction corresponding to the points a-f in Figure 4 are shown in Figure 5(a–f), respectively. The left parts are the solutions while the rights are the electrodes. Parts in between are the interfaces. The color variation appears evidently at the interface, where turning yellow means the increase of the OPL while turning red means the sharp increase. The larger the value of the OPL is, the more evident the color shows. The green area in those figures indicates that the difference of OPL is nearly constant. Since there is nearly no change at the interface in Figure 5(a), the parts corresponding to the electrolyte, electrode, and interface have been indicated in Figure 5(a) so they can be read more easily. The locations of the interfaces in Figure 5(b–f) are the same as Figure 5(a). This also applies for Figure 7. As can be seen, a yellow color appears only at the interface, showing that the OPL was rather stable in most of the bulk solution, but it increases at the metal/solution interface, which indicated the iron electrode dissolved as ions during the reactions. At points a and b, the change of the OPL was hardly detectable when the current density is relatively small, as shown in Figure 5(a,b). respectively. As time goes on, the OPL increases at points c and d (Figure 5(c,d)), indicating the concentration of metal ions increases sharply when the current density grows up. The concentration decreases when the density drops (Figure 5(e,f)). Since the OPL distribution is nearly uniform, there is an approximate one-dimensional mass transport at the metal/solution interface, which is consistent with the general corrosion processes of iron in Na_2_SO_4_ solution.

**Figure 6.**
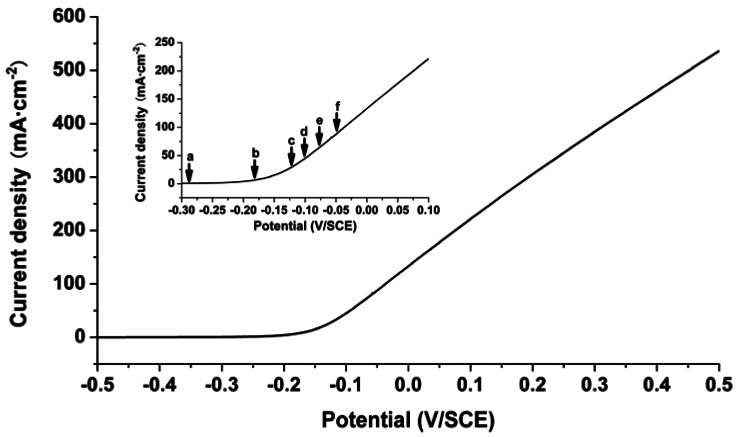
Potentiodynamic polarization curve of the iron electrode in 0.5 M NaCl solution at 10 mV·s^−1^.

**Figure 7.**
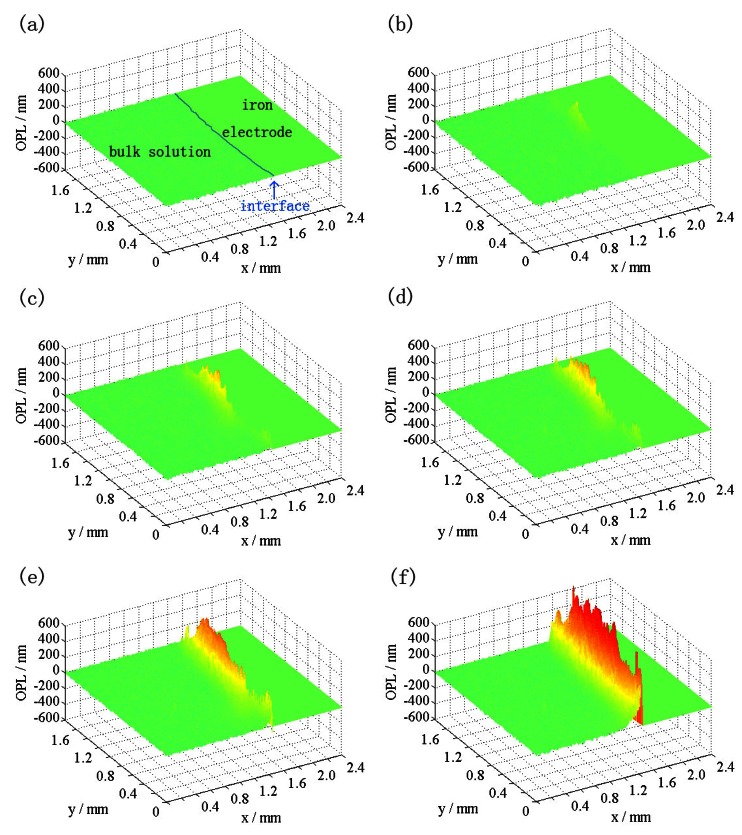
The distributions of the OPL difference at different times in comparison with the initial time during the process of the iron electrode in 0.5 M NaCl solution at 10 mV·s^−1^.

The potentiodynamic sweep curve of the iron electrode in 0.5 M NaCl solution at 10 mV·s^−1^ is illustrated in Figure 6. It is widely accepted that localized corrosion appears in aggressive environments, especially in media containing chloride ions. When the potential is more positive than −0.20 V *vs*. SCE, the current evidently grows up with the increase of potential. Figure 7 displays the distributions of the difference of OPL at the time points in Figure 6. At the initial of the sweep, the concentration change at the interface is hardly detectable (Figure 7(a)). With the increase of the current density, the appearance of a yellow area in Figure 7(b) indicates that the formation of Fe^2+^ ions increases the OPL at the local area, and localized corrosion appears at the iron/solution interface. In Figure 7(c), the former yellow area enlarges and another yellow area appears which can be seen more clearly in Figure 7(d,e). In contrast to the one-dimensional mass transport of general corrosion in Na_2_SO_4_ solution, localized corrosion of the iron electrode formed under the presence of chloride ions. As time goes on, the OPL becomes larger and larger (Figure 7(e,f)). Many more metal ions have been produced and transported out. The whole iron electrode becomes active and the distribution of the OPL difference becomes more uniform at the metal/solution interface (Figure 7(f)).

The thickness of diffusion layer can be visually presented in the OPL distributions. In the case of the potentiodynamic sweep of the iron electrode in 0.5 M Na_2_SO_4_, the thickness of diffusion layer increased with the rise of the current density, and then decreased with the drop. In 0.5 M NaCl, the diffusion layer at the iron/solution interface was not uniform when the localized corrosion appeared. Compared to published work of the authors [11], the results presented here are more revealing. Moreover, digital interferograms are processed automatically to realize consecutive observation of corrosion processes and the dynamic changes of OPL are quantitatively measured to provide a direct evidence of localized corrosion.

## 5. Conclusions

A Mach-Zehnder interferometer has been employed to visualize the localized processes at the metal/solution during the electrochemical process. In the case of the potentiodynamic sweep of the iron electrode in 0.5 M Na_2_SO_4_ and 0.5 M NaCl solutions, the distributions of the OPL difference show general and localized corrosion during the electrochemical reactions, respectively. The changes of OPL in the object beam can help capture otherwise inconspicuous information in specimens for quantitative measurement. This method can detect a minimal OPL difference of about 10 nm caused by the electrochemical reactions. It is proved to be sensitive enough to detect localized corrosion at the metal/solution interface. Although its maximum transverse resolution is confined by diffraction limits, it can provide *in situ* observation of the dynamic processes and study localized corrosion in micro-scale systems. It can reflect the corrosion rate but cannot provide the number and distribution of pits. Further efforts will be made to obtain the quantitative measurement of corrosion rate.
